# Reducing Temperatures in Fever

**Published:** 1894-12

**Authors:** 


### HOMŒOPATHIC PHYSICIAN,

A MONTHLY JOURNAL OF

HOMŒOPATHIC MATERIA MEDICA AND CLINICAL MEDICINE.

"If our school ever gives up the strict inductive method of Hahnemann, we are lost, and deserve only to be mentioned as a caricature in the history of medicine."—CONSTANTINE HERING.

Vol. XIV. DECEMBER, 1894. No. 12.

### EDITORIAL.

REDUCING TEMPERATURES IN FEVER.—One of the most important problems before the old school of medicine at the present time is the reduction of the high temperatures occurring in the course of acute diseases.

The prevailing opinion among these physicians is that the chief danger to the patient in such diseases lies in the high temperature. Fixing their attention upon the question of fever, heroic attempts are made to force down or suppress this manifestation of heat. Their efforts in this direction have, from time to time, become always more and more heroic until they now reach an extreme which, however successful they may be in reducing the absolute height of the thermometer, are nevertheless exceedingly detrimental to the general welfare of the patient.

From Quinine to Antipyrine, Antifebrine and other similar drugs for forcibly producing a suppression, they have advanced to the full bath.

Ingenuity has been taxed to produce the best method of applying this agent, and we have the most remarkable devices for accomplishing the result. Thus, one physician treats his cases of typhoid fever by bringing into the patient's chamber a bath-tub that resembles somewhat a coffin, into which the patient is placed and his body kept submerged, all except his head, of course, in water of a temperature of 90 to 98 degrees, for a period varying from six to thirty-one days! A very clear account of this treatment may be found in the *Medical Annual* for 1891, published by E. B. Treat, No. 5 Cooper Union, New York, at page 471.

Now a reaction against this mania for reducing temperature has set in, and the question is being asked whether it is right to suppresss the pyrexia. *The Homœopathic World* for October, in an editorial entitled, "Knocking Down Temperatures or Knocking Down Patients," discusses this question especially with reference to the administration of massive doses of "Antipyretic drugs." Incidentally it quotes from an allopathic journal whose editor doubts the wisdom of these heroic measures. This editor is reported as saying that " more recent observations would seem to indicate that we should view fever in the light of a willing ally against the marauding and ubiquitous bacillus with its train of excrementitial toxines."

The error of this kind of treatment is, of course, to be found in the fixing of the attention too closely upon one symptom in the case, almost to the exclusion of the others. One " view " is adopted only to be displaced by another, and none being guided by the knowledge of any fixed law. Homœopathists are naturally, by reason of their knowledge of law, better equipped for dealing with fever. Yet they need to be admonished even in this regard, for they, too, are found to be following the error of paying excessive attention to the one symptom of fever, as was shown in the editorial on "Aconite" in the September number of this journal.

For this reason these remarks are here introduced that the followers of Hahnemann may more vividly realize the mistake they are making.

It cannot too often be repeated that the true physician should avoid acting upon any kind of " views " or opinions in making his prescriptions, but should set aside all " pride of intellect," and thus avoiding all follies of speculation, faithfully apply the law of the similars, and industriously compare the symptoms of the case with the symptomatology of the drugs in the materia medica. As has been so frequently said before in these pages the discovery of the true simillimum in any given case will give the highest measure of success, while neglect of this course must end in failure.

